# Improving Sorbents for Glycerol Capture in Biodiesel Refinement

**DOI:** 10.3390/ma10060682

**Published:** 2017-06-21

**Authors:** Brandy J. Johnson, Brian J. Melde, Martin H. Moore, Anthony P. Malanoski, Jenna R. Taft

**Affiliations:** 1 Center for Bio/Molecular Science and Engineering, Naval Research Laboratory, Washington, DC 20375, USA; brian.melde@nrl.navy.mil (B.J.M.); martin.moore@nrl.navy.mil (M.H.M.); anthony.malanoski@nrl.navy.mil (A.P.M.); 2 Formerly, NOVA Research Incorporated, Alexandria, VA 22308, USA; jennartaft@gmail.com

**Keywords:** biodiesel, solid phase extraction, organosilicate, sorbent

## Abstract

Biodiesel is produced by transesterification of animal fat, vegetable oil, or waste cooking oil with alcohol. After production costs, the economic viability of biodiesel is dependent on what steps are necessary to remove impurities following synthesis and the effectiveness of quality control analysis. Solid-phase extraction offers a potentially advantageous approach in biodiesel processing applications. Nanoporous scaffolds were investigated for adsorption of glycerol, a side product of biodiesel synthesis that is detrimental to engine combustion when present. Materials were synthesized with varying pore wall composition, including ethane and diethylbenzene bridging groups, and sulfonated to promote hydrogen bonding interactions with glycerol. Materials bearing sulfonate groups throughout the scaffold walls as well as those post-synthetically grafted onto the surfaces show notably superior performance for uptake of glycerol. The sorbents are effective when used in biodiesel mixtures, removing greater than 90% of glycerol from a biodiesel preparation.

## 1. Introduction

Biodiesel is produced by transesterification of animal fat, vegetable oil, or waste cooking oil with an alcohol. A number of studies have focused on evaluating the economic viability of biodiesel [1,2,3,4]. Production costs are approximately 0.5 USD/L, 1.5 times that of petroleum-based diesel, with virgin feedstocks comprising 50 to 70% of costs [2,3]. The feedstock costs can be reduced through the use of waste cooking oils and similar products, but these materials increase the complexity of synthesis and, often, the presence of contaminants [4]. In addition to providing a renewable source of energy, biodiesel is attractive as an alternative fuel due to related reduced environmental impact; use of biofuels decreases emission of atmospheric pollutants [5]. Procurement of water together with liquid and solid waste disposal comprise up to 10% of manufacturing costs [2,3] while also influencing the environmental impact of this alternative fuel source. As an example, multiple wash steps for biodiesel purification can generate gallons of waste water for each gallon of usable biofuel. Biodiesel contaminants include glycerol, alcohols, free fatty acids, surfactants, and residual catalyst. Glycerol is a significant contaminant in biodiesel, but it can be recovered and purified to aid in offsetting the costs of biodiesel production [3].

Currently, dry wash media use is largely limited to small operations and hobbyists. These media can be used for biodiesel contaminant removal without excessive waste water production. This approach also reduces production time and requires less space when compared to water washing. BD ZorbX and DudaLite (DW-R10^®^) ion exchange resin are commercial examples of these types of materials. BD ZorbX is a mixture of cellulose fibers from hardwood intended to absorb a variety of contaminants including surfactants, glycerol, and catalyst; Eco2Pure™ is a similar material. DW-R10 is intended as a polishing step following bulk glycerol removal. Claims for this sorbent are removal of surfactants, glycerol, catalyst, and water. Drionex, Purolite^®^ PD206, and Magnesol D-Sol are intended for similar applications [6].

Nanoporous scaffolds within a macrotextured structure offer an alternative to currently available commercial dry wash media. The materials under consideration for this study are hybrid inorganic–organic materials [7,8,9,10,11,12,13,14,15,16,17] in which inorganic and organic moieties are mixed on the molecular scale through the use of bridged polysilsesquioxane precursors [18]. The materials offer large macropores with a templated mesopore structure [7,12,17,19]. While a number of applications have been reported for mesoporous sorbents, additional applications can be facilitated through the use of these hierarchical structures; the macroscale features reduce diffusion limitations and increase access to the mesopore volumes [20,21]. We have previously described materials of this type for the capture of nitroenergetic and organophosphate targets [20,22]. The materials of the current study were developed for adsorption and removal of glycerol, which can result in fuel tank deposits, injector corrosion, and higher emissions of aldehydes. The materials are synthesized with varying pore wall composition then sulfonated to promote hydrogen bonding interactions with glycerol. While sulfonation of the material provides a similar functionality to that present in several of the commercial dry wash media mentioned above, incorporation of the sulfonate groups on and within the scaffold and adjacent to varying structures provides an opportunity to evaluate the potential for improving sorbent performance through scaffold tailoring.

## 2. Results

### 2.1. Sorbents

**Figure 1:**
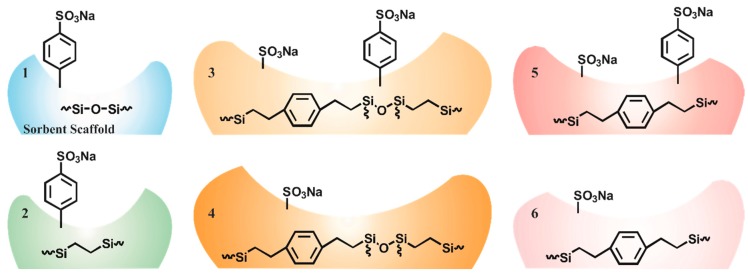
Glycerol sorbents. Schematics of the material variations are presented here showing both the groups within the sorbent scaffold (shaded area) and the groups added during post-synthesis processing. The numbers identify the type (1 through 6) and are used in the text to describe the sorbents as well as in the sorbent names. For example, S65-Ph(1) is a Type 1 sorbent while E50-Ph(2) is a Type 2 sorbent.

A previous study by Chen et al. utilized sulfonation of a poly-(styrene-co-divinylbenzene) resin to increase hydrogen bonding with the material and binding of glycerol by the material [23]. The study did not consider the impact of the scaffold or the morphology of the resin on target adsorption. Because the chemical structures in proximity to the sulfonate groups as well as the density of the sulfonate groups is likely to impact the interaction of those groups with glycerol, a series of porous organosilicate materials were synthesized in which the pore wall composition was varied (Table 1). In some cases, S65, S85, E25, and E50, all sulfonated sites were included through pendant phenyl groups added to the materials following surfactant extraction; no sites for sulfonation were included in the base sorbent. In other cases, ED11 and D13, diethyl benzene groups comprise a portion of the scaffold walls. Sulfonation of the materials directly (ED11a(4) and D13a,b,c(6)) or sulfonation following grafting of additional phenyl groups was then completed. The schematic provided in Figure 1 illustrates the intended variations in the resulting materials (see also Table 1).

**Figure 2:**
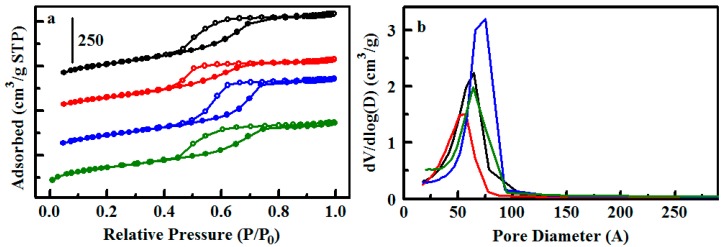
Nitrogen sorption analysis for sulfonated sorbents. (**a**) Nitrogen adsorption/desorption isotherms for S65 (black), S65-Ph(1) (red), S85 (blue), and S85-Ph(1) (green); (**b**) Pore size distributions for the sorbents colored as in (**a**).

As shown in Table 2, the various synthetic procedures lead to a variety of morphological characteristics. The Type 1 materials (Figure 1) provide a sodium-exchanged sulfonated phenyl group pendant on a silicate scaffold. Nitrogen sorption experiments on S65-Ph(1) and S85-Ph(1) yield type IV isotherms characteristic of mesoporous materials, showing steep increases in nitrogen adsorbed in the mid-pressure region (*P*/*P*_0_ ca. 0.4–0.7) corresponding to capillary condensation in mesopores (Figure 2). The silicate scaffolds have ordered mesostructures in which cylindrical mesopores are arranged with 2D-hexagonal packing; the pore sizes of 56 Å (S65-Ph(1)) and 64 Å (S85-Ph(1)) are influenced by the amounts of mesitylene, the micelle swelling agent, included in their syntheses [24,25].

**Figure 3:**
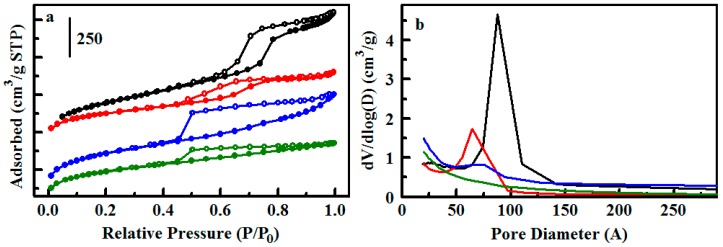
Nitrogen sorption analysis for ethane bridged sorbents. (**a**) Nitrogen adsorption/desorption isotherms for E25 (black), E25-Ph(2) (red), E50 (blue), and E50-Ph(2) (green); (**b**) Pore size distributions for the sorbents colored as in (**a**).

Type 2 materials provide the same pendant functional group on an ethane-bridged organosilicate sorbent. The base E50 scaffold has been functionalized and studied previously for adsorption applications [26]. It has a combination of disordered micropores and mesopores, evidenced in the adsorption branch of its nitrogen sorption isotherm by an initial increase in the low relative pressure region and gradual increase throughout the mid and high relative pressure regions (Figure 3). No defined peak is observed in the pore size distribution of E50-Ph(2) after functionalization (Figure 3); an indistinct feature ca. 76 Å is present in the base material. E25, synthesized with half the amount of mesitylene used for E50, does produce a distinct pore size distribution peak at 64 Å following functionalization (Figure 3). The type IV nitrogen sorption isotherm shape with steep adsorption step in the mid relative pressure region is similar to those of ordered S65-Ph(1) and S85-Ph(1). The E25-Ph(2) and E50-Ph(2) products have the highest surface areas (>800 m^2^/g) and pore volumes (>0.6 cm^3^/g) of the sorbents used in this study.

**Figure 4:**
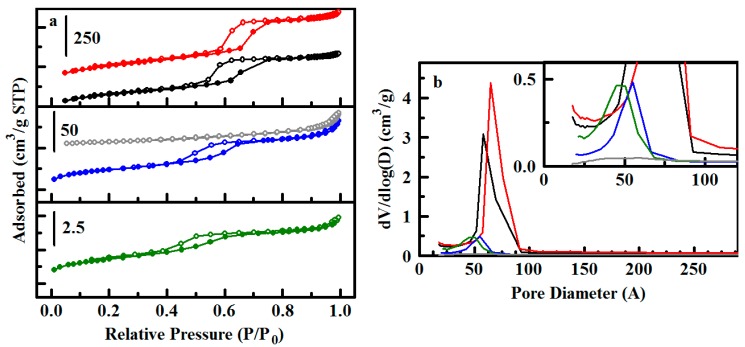
Nitrogen sorption analysis for sulfonated sorbents. (**a**) Nitrogen adsorption/desorption isotherms for ED11 (black), ED11-Ph(3) (red), ED11a(4) (blue), ED11-Ph1(3) (green), and ED11-Ph2(3) (gray); (**b**) Pore size distributions for the sorbents colored as in (**a**). Inset provides a zoomed view of the region up to 115 Å.

Type 3 materials based on ED11 have 50% ethane bridging groups with the remainder comprised of diethylbenzene groups, allowing for direct sulfonation of phenylene groups within the pore walls, as well as the grafted phenyl groups. Type 4 refers to sodium-exchanged sulfonated ED11 with no grafted phenyl moieties (ED11a(4)). Direct functionalization of the scaffold walls leads to notably low values for surface area and pore volume (Table 2), despite the base ED11 material having values ca. 700 m^2^/g and 0.8 cm^3^/g. Most products sulfonated with undiluted H_2_SO_4_ display Type II nitrogen sorption isotherms indicative of relatively non-porous materials (e.g., ED11-Ph2(3) in Figure 4).

**Figure 5:**
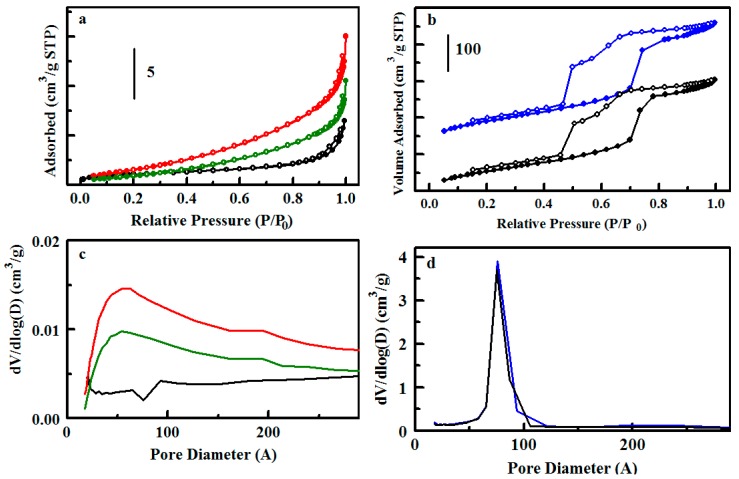
Nitrogen sorption analysis for sulfonated sorbents. (**a**) Nitrogen adsorption/desorption isotherms for D13-Ph1(5) (black), D13a(6) (red), and D13c(6) (green); (**b**) Nitrogen adsorption/desorption isotherms for D13-Ph2(5) (black) and D13b(6) (blue); (**c**,**d**) Pore size distributions for the sorbents colored as in (**a**,**b**), respectively.

Types 5 and 6 sorbents based on D13 have the highest concentration of diethylbenzene bridging groups in their scaffold walls, being synthesized with bis(trimethoxysilylethyl)benzene as the sole silane precursor. Type 5 also includes grafted pendant phenyl moieties. The base D13 scaffold exhibits impressive porosity characteristics for a templated material synthesized from this relatively flexible bridged-silane, with a surface area ca. 420 m^2^/g and pore size distribution peak at 74 Å. Functionalization using undiluted H_2_SO_4_ collapses the mesostructure, as shown by the nitrogen sorption isotherms and pore size distributions (Figure 5). D13b(6) was treated with 50% H_2_SO_4_, which preserved mesoporosity (Figure 5). The nitrogen isotherm shows considerable hysteresis between its adsorption and desorption branches, indicative of “bottle-necks” in the mesostructure.

### 2.2. Glycerol Capture from Aqueous Solution

**Figure 6:**
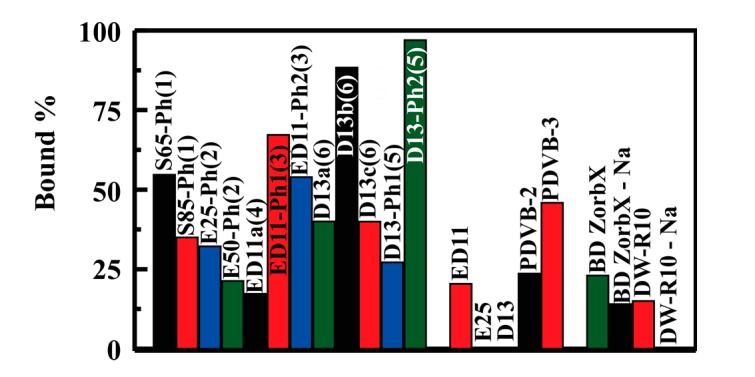
Glycerol binding by the sorbents evaluated from identical batch type experiments using 271 μM glycerol (10 mL) with 15 mg sorbent.

**Figure 7:**
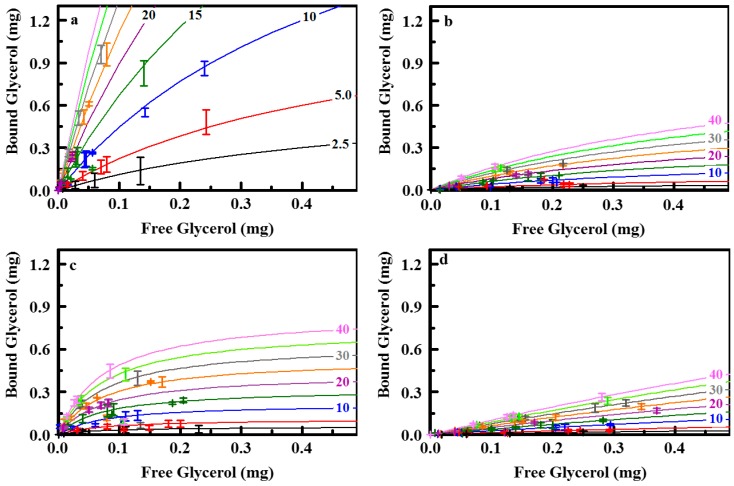
Glycerol binding data and Langmuir-Freundlich isotherms for D13b(6) (**a**), S85-Ph(1) (**b**), ED11-Ph(3) (**c**), and ED11 (**d**), sorbent with no sulfonation. Isotherms for 2.5, 5.0, 10, 15, 20, 25, 30, 35, and 40 mg presented.

Batch type experiments were used for comparison of sorbent variants with regard to their potential for capture of glycerol from water. Analysis was completed by determination of the glycerol remaining in solution following overnight incubation of varying sorbent masses with glycerol solutions of varied concentration. Analysis of glycerol was based on a modification of the ASTM D6584-13 method [27]. Figure 6 provides single point comparison of glycerol binding by the synthesized sorbents. As shown, under identical conditions, the D13-Ph2(5) variant (Type 6) bound more target on a per mass basis than all other sorbents including the commercially available materials. D13b(6) and the Type 3 materials also stand out, with greater performance than other sorbents. Complete isotherms were generated for more thorough comparison of the materials. Figure 7 provides examples of the binding isotherms collected; additional isotherms are included in the Supporting Information, Figures S1–S5.

We have shown previously that binding of targets by these sorbents can be described by a phenomenological model such as the Langmuir-Freundlich formulation [20,28,29],

(1) $$q=\frac{q_{s}AkL^{n}}{1+kL^{n}}$$

where *q* is the amount of target bound per unit sorbent surface area, *L* is the free ligand, *k* is the affinity coefficient, *n* is the homogeneity factor, and *A* is the total sorbent surface area. The typically used saturation capacity is *q_s_*. Table 2 provides a summary of the calculated parameters. The dynamic range of the analytical method utilized for these studies limits the range of target concentrations that can be interrogated. Due to this limited range, it was necessary to access a series of isotherms at different sorbent masses to obtain a data set that could be fit with confidence. Sorbent masses were varied from 2.5 to 50 mg in a fixed solution volume (20 mL). This variation is captured by the area (*A*) in Equation (1). All data not representing binding of 0 or 100% of target was used in the generation of the isotherm for a given sorbent material.

ED11, with ethane and diethylbenzene bridging groups, and E25, with ethane bridging groups, sorbents provide points for comparison of the sulfonated materials. There was no significant binding of glycerol by the E25 sorbent. ED11 bound some glycerol but less than any of the sulfonated sorbents (Figure 6). The addition of pendant sulfonatophenyl groups (E25-Ph(2)) dramatically increased capture of glycerol by E25. The similar sorbent, E50-Ph(2), showed a lower glycerol saturation capacity (see isotherms for E25-Ph(2) and E50-Ph(2) in the Supporting Information, Figure S1). The E50-Ph(2) sorbent shows a broad pore size distribution with no defined peak and an increased percentage of pores at smaller diameters while the E50 sorbent had a defined peak around 76 Å (Figure 3). It is likely that the smaller saturation capacity of the E50-Ph(2) material is related to inhibited access to the total surface area of the sorbent owing to collapsed or restricted pores. The capacity could be impacted by incomplete sulfonation of the total surface area; however, the affinity of the sorbent for the target is higher than that of the E25-Ph(2) sorbent. Saturation capacities for the binding of glycerol by TEOS based sorbents with pendant sulfonatophenyl groups (S65-Ph(1) and S85-Ph(1)) were greater than those of the ethane-bridge sorbents while affinity coefficients were lower. These sorbents retained the pore structure of the base sorbents to a greater degree following functionalization.

Sulfonation of the sorbent synthesized with a mixture of ethane and diethylbenzene bridging groups (ED11a(4); 18.0 mg/g) did not yield an increase in per mass binding when compared to the base sorbent (ED11; 38.8 mg/g). It is likely that pore collapse contributes significantly to the lack of capacity observed for the ED11a(4) variant; specific surface area is reduced from 478 m^2^/g to 87 m^2^/g following sulfuric acid treatment. Parameters from fitting the isotherm indicate greater affinity in the sulfonated sorbent. When considered on the basis of surface area, the saturation capacities are 81 μg/m^2^ for ED11 and 207 μg/m^2^ for ED11a(4). Addition of pendant sulfonatophenyl groups (ED11-Ph2(3)) to this sorbent resulted in a distinct improvement in per surface area glycerol binding (452 μg/m^2^) with further reduced per mass capacity (12.2 mg/g) associated with significant additional loss in surface area (27 m^2^/g).

The tradeoff between pore collapse and per surface area saturation capacities is further illustrated by the D13 materials. D13b(6) retains 337 m^2^/g following treatment with sulfuric acid; the per surface area binding capacity of this material is six times less than that of D13a(6) or D13c(6) (~3 m^2^/g). This inverse relationship would tend to indicate that more complete sulfonation of the scaffold provides greater capacity, as expected; however, the cost of sorbent materials is driven by the mass of the sorbent, not the total surface area. Because the cost of materials is related to the mass utilized in applications, we have reported both per mass and per surface area capacities in Table 2, facilitating comparison of the materials by both metrics. Adjustment of the sulfonation conditions for the material incorporating pendant phenyl groups on the diethylbenzene bridged material (D13-Ph2(5)) produced a sorbent that retained much of the initial morphological characteristic as well as a high degree of sulfonation. This sorbent provided the highest per mass saturation capacities of the series (552 mg/g) with an affinity coefficient similar to those of the collapsed D13 structures (4.15 mg^−1^). The per surface area saturation capacity for D13-Ph2(5) is more than double that of the D13b(6) sorbent (1780 μg/m^2^ versus 810 μg/m^2^).

**Figure 8:**
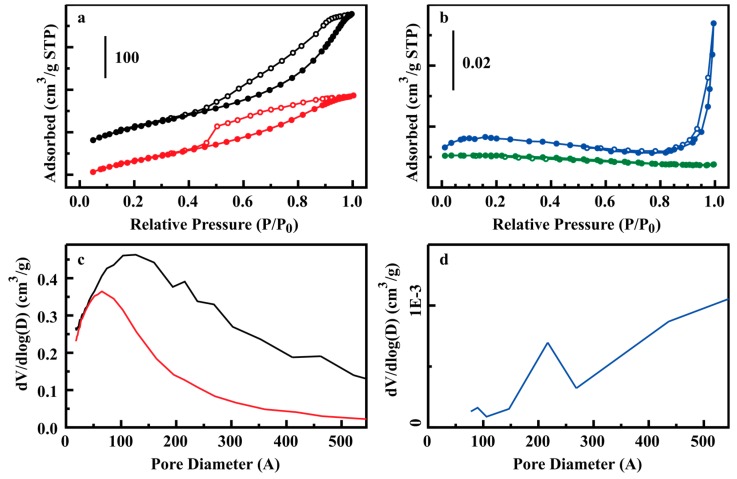
Nitrogen sorption analysis for divinylbenzene resins and commercial sorbents. (**a**) Nitrogen adsorption/desorption isotherms for PDVB-2 (black) and PDVB-3 (red); (**b**) Nitrogen adsorption/desorption isotherms for BD ZorbX (blue), and DW-R10 (green); (**c**,**d**) Pore size distributions for the sorbents colored as in (**a**,**b**), respectively. Nitrogen adsorption by DW-R10 was insufficient for pore diameter analysis.

The binding of glycerol from aqueous solution by the sorbents developed under this study was compared to the commercially available sorbents BD Zorbx and DW-R10. Additional points of comparison were desired; however, repeated attempts to obtain Amberlite™ BD10DRY (Dow Chemical Company, Midland, MI, USA) were unsuccessful. Amberlite gel-type ion-exchange resins are based on a styrene divinylbenzene copolymer backbone. With this in mind, two mesoporous poly(divinylbenzenes) were adapted from the literature [30] and sulfonated for comparison to the silicate based materials (Section 3.5; Figure 8). Fitting of the glycerol binding result for these PDVB resins resulted in saturation capacities of 4.7 and 11.2 mg/g for PDVB-2 and PDVB-3, respectively (Table 2). Isotherms are provided in the Supporting Information, Figure S5. Both PDVB resins outperformed the commercially obtained sorbents (Table 2). The Types 3–6 materials, with the exception of D13-Ph1(5), outperformed the PDVB resins under both per mass and per area comparisons. PDVB-3 had a higher saturation capacity than D13-Ph1(5) when compared on a per mass basis.

### 2.3. Glycerol Capture from Biodiesel

**Figure 9:**
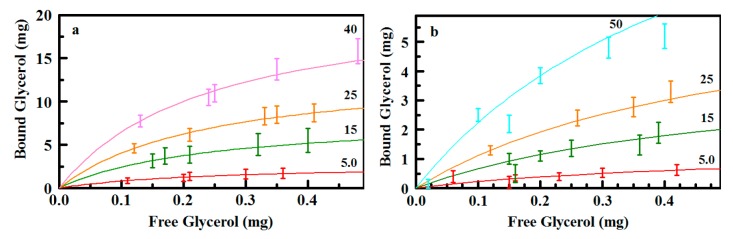
Glycerol binding data for D13-Ph2(5) (**a**) and D13b(6) (**b**) from biodiesel. Isotherms (lines) are from data sets collected in aqueous solution (Table 2).

Capture of glycerol from biodiesel based solutions was also evaluated (see Section 3.7). Biodiesel was spiked with a known concentration of glycerol. Figure 9 presents representative data for capture of glycerol from this solution (additional results, Supporting Information, Figure S6 through S9). The base ED11 sorbent (no sulfonation) did not remove glycerol from the biodiesel solution. Though the data sets tend to be noisier than those collected in aqueous solution (Figure 8), capture of glycerol by the sulfonated ED11 and D13 based sorbents (Types 4 and 6) was similar to that observed for capture from aqueous solution. Capture of glycerol by the S65-Ph(1) sorbent was less than that from aqueous solution. It is possible that this is a result of hydrophilic/hydrophobic interactions as the backbone of this sorbent would tend to be more hydrophilic than those of the others in the series.

**Figure 10:**
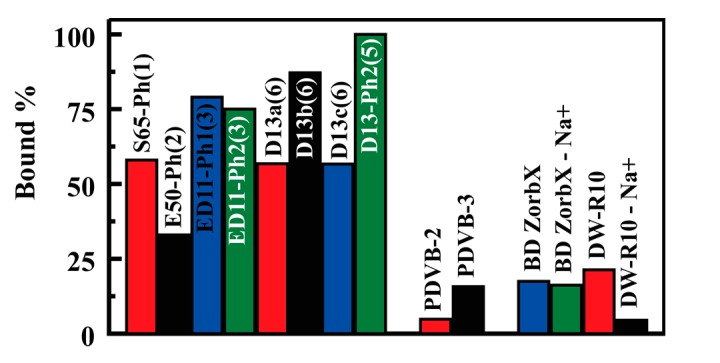
Glycerol binding from biodiesel from identical batch type experiments using 250 μM glycerol (10 mL) with 30 mg sorbent.

Figure 10 provides single point comparison of glycerol capture from biodiesel by developed sorbents, the commercial resins, and the synthesized poly(divinylbenzene) resins (PDVB-2 and PDVB-3). We have also included data for sodium exchanged versions of the commercial media. D13-Ph2(5), a Type 5 sorbent, dramatically out performs the PDVB resins and the commercial sorbents, binding 100% of glycerol from the challenge solution. As in the aqueous study, D13b(6) binds more target than the lower surface area D13a(6) and D13c(6) sorbents. This pattern holds for the two Type 3 sorbents based on ED11-Ph(3).

## 3. Materials and Methods

Bis(trimethoxysilylethyl)benzene (DEB) and 1,2-bis(trimethoxysilyl)ethane (BTE) were obtained from Gelest, Inc. (Tullytown, PA, USA). Tetramethyl orthosilicate (98%), sodium hydroxide, methanol, hydrochloric acid, mesitylene (1,3,5-trimethylbenzene or TMB), Pluronic^®^P123, and glycerol (56-81-5) were purchased from Sigma-Aldrich (St. Louis, MO, USA). Water was deionized to 18.2 MΩ cm using a Millipore Milli Q UV-Plus water purification system. Biodiesel was produced (details in Section 3.7) in small batches using fresh canola oil purchased from a local supermarket.

### 3.1. Synthesis of E25-Ph(2) and E50-Ph(2) (Type 2, Ethane-Bridged Organosilicates, Ph Indicates Grafted Phenyl Groups)

Scaffold materials were synthesized as described in previous reports [21,26]. Pluronic P123 (3.8 g) and 0.5 g of mesitylene (E25) or 1.0 g of mesitylene (E50) were dissolved in 12.14 g of 0.1 M HNO_3_ with heat (~65 °C) and magnetic stirring. The stirring mixture was allowed to cool to room temperature before drop-wise addition of 4.24 g of 1,2-bis(trimethoxysilyl)ethane. The mixture was stirred to homogenize (~1 min) and transferred to culture tubes. Tubes were tightly sealed and incubated in an oven at 60 °C overnight. The tubes were unsealed, and the white monolithic gels were heated for 2 d at 60 °C followed by 2 d at 80 °C. The block copolymer (Pluronic P123) was extracted from the dried materials by refluxing over-night in 1 M HCl in ethanol. The solid was collected by vacuum filtration. This process was repeated two more times. The collected solid was washed thoroughly with ethanol and water and dried at 110 °C.

Grafting with phenyl groups followed a simple published procedure, beginning with an additional drying step at >100 °C in a vacuum [31]. Sorbent (1.0 g) was refluxed with 5 mL of phenyltriethoxysilane in 50 mL of toluene for 24 h. Grafted material was collected by gravity filtration, washed thoroughly with ethanol, and dried at 110 °C. Sulfonation was accomplished as described in the literature by magnetically stirring the phenyl-grafted material in 25 mL of H_2_SO_4_ at 75 °C for 1 d [32]. The acidic mixture was then added to ≥200 mL of H_2_O. Solid was collected by gravity filtration, washed with H_2_O, and dried at 110 °C. Sulfonated material was magnetically stirred in 50 mL of 1 M NaCl solution at RT for 1 d followed by collection by gravity filtration, washing with H_2_O, and drying at 110 °C. The final sulfonated, phenyl grafted materials are referred to as E25-Ph(2) and E50-Ph(2), reflecting their base E25 or E50 scaffold, the phenyl group modification (Ph), and the sorbent type (2).

### 3.2. Synthesis of ED11-Ph1(3), ED11-Ph2(3), and ED11a(4) (Types 3 and 4, Mixed Ethane and Diethylbenzene-Bridged Organosilicates, Ph Indicates Grafted Phenyl Groups)

The base scaffold (ED11) was synthesized as previously reported [28]. Pluronic P123 (3.8 g) and mesitylene (1.0 g) were dissolved in 15.0 g of 0.1 M HNO_3_ with heat (~65 °C) and magnetic stirring. The stirring mixture was then allowed to cool to room temperature. A silane mixture of 1,2-bis(trimethoxysilyl)ethane (2.12 g) and bis(trimethoxysilylethyl)benzene (2.94 g) was added drop-wise, and the mixture was stirred to homogenize (~1 min). The mixture was transferred to culture tubes that were tightly sealed and incubated in an oven at 60 °C overnight. The tubes were unsealed, and the white monolithic gels were heated for 2 d at 60 °C followed by 2 d at 80 °C. The block copolymer was extracted from the dried materials by refluxing overnight in 1 M HCl in ethanol; the solid was collected by vacuum filtration. This process was repeated two more times. The collected solid was washed thoroughly with ethanol and water, then dried at 110 °C. Grafting, sulfonation, and sodium exchange for ED11-Ph1(3) and ED11-Ph2(3) were completed using the protocols described in Section 3.1; ED11-Ph1(3) and ED11-Ph2(3) refer to similarly but separately synthesized materials. The sulfonation step produces the variation in material characteristics. ED11a(4) was not grafted with phenyl groups prior to sulfonation and sodium exchange. The final sulfonated (Type 4) and sulfonated, phenyl grafted (Type 3) materials are referred to as ED11a(4), ED11-Ph1(3), and ED11-Ph2(3), reflecting their base ED11 scaffold, the phenyl group modification (Ph), and the sorbent type (3 or 4).

### 3.3. Synthesis of S65-Ph(1) and S85-Ph(1) (Type 1, silicates, Ph Indicates Grafted Phenyl Groups)

Scaffold materials were synthesized based on a previously published procedure [24,25]. Pluronic P123 (4.0 g) and 0.65 g (S65) or 0.85 g (S85) mesitylene were dissolved in 12.0 g of 1.0 M HNO_3_ with heat (~65 °C) and magnetic stirring. The mixture was allowed to cool to room temperature before drop-wise addition of 5.15 g tetramethyl orthosilicate, and the mixture was stirred to homogenize (~1 min). The mixture was transferred to culture tubes that were tightly sealed and incubated at 60 °C overnight. The tubes were unsealed, and the white monolithic gels were heated at 60 °C for 6 d. The material was calcined under ambient atmosphere: temperature was ramped 1 °C/min to 650 °C and held for 5 h. Material was refluxed in 1 M HCl for 1 d, similar to the solvent extraction process for other materials, to condition the surface similarly for grafting. It was then collected by vacuum filtration, washed with ethanol and H_2_O, and dried at 110 °C. Grafting, sulfonation, and sodium exchange for these sorbents were completed using the protocols described in Section 3.1. The final sulfonated, phenyl grafted materials are referred to as S65-Ph(1) and S85-Ph(1), reflecting their base S65 or S85 scaffold, the phenyl group modification (Ph), and the sorbent type (1).

### 3.4. Synthesis of D13a(6), D13b(6), D13c(6), D13-Ph1(5), and D13-Ph2(5) (Types 5 and 6, Diethylbenzene-Bridged Organosilicates, Ph Indicates Grafted Phenyl Groups) 

Scaffold materials were synthesized by dissolving Pluronic P123 (3.8 g) and mesitylene (1.8 g) in 16.0 g of 0.1 M HNO_3_ with heat (~65 °C) and magnetic stirring. The mixture was allowed to cool to room temperature prior to drop-wise addition of bis(trimethoxysilylethyl)benzene (5.87 g), and the mixture was stirred to homogenize (~1 min). The mixture was transferred to culture tubes that were tightly sealed and incubated in an oven at 60 °C overnight. The tubes were unsealed, and the white monolithic gels were heated for 2 d at 60 °C and 2 d at 80 °C. Block copolymer was extracted from the dried materials by refluxing overnight in 1 M HCl in ethanol; the solid was collected by vacuum filtration. This process was repeated two more times. The collected solid was washed thoroughly with ethanol and water, then dried at 110 °C. Grafting, sulfonation, and sodium exchange for D13-Ph1(5) and D13-Ph2(5) were completed using the protocols described in Section 3.1. D13a(6), D13b(6), and D13c(6) were not grafted with phenyl groups prior to sulfonation and sodium exchange. The designations a, b, and c refer to similarly but separately synthesized materials. D13b(6), in particular, was sulfonated using 50% H_2_SO_4_ rather than undiluted acid. The final sulfonated (Type 6) or sulfonated, phenyl grafted (Type 5) materials are identified by their base scaffold (D13a, D13b, etc.), the phenyl group modification (Ph), and the sorbent type (5 or 6).

### 3.5. Synthesis of PDVB-2 and PDVB-3 (Poly(Divinylbenzene) Resins)

Divinylbenzene (DVB) resins were synthesized in a Teflon-lined autoclave reactor (23 mL capacity) [30]. For PDVB-2, DVB (1.5 g; Aldrich technical grade, 80%) was added to tetrahydrofuran (15 mL) with water (0.75 mL) and 2,-2′-azobis(2-methylpropionitrile) (0.0375 g; Aldrich, 98%). The reactor was heated at 100 °C for 48 h. Following cooling to room temperature, the reactor was opened and the resin was allowed to dry overnight at room temperature followed by complete drying at 100 °C. PDVB-3 was synthesized identically with the exception of the water co-porogen used in the PDVB-2 synthesis, which was omitted. Ground resin was added to hexane (20 mL) to produce a wet slurry, and sulfuric acid was added (25 mL) for the sulfonation step. The mixture was heated at 75 °C for 1 d before addition of water (300 mL). Sulfonated material was collected by vacuum filtration and washed thoroughly with water before drying at 100 °C. Sodium exchange was completed by stirring the material in 1 M NaCl (50 mL) at room temperature for 1 d. The resin was then washed with water, collected by vacuum filtration, and dried at 100 °C. Sodium exchange for commercial sorbents was completed in the same manner.

### 3.6. Characterization

Nitrogen adsorption-desorption analysis was performed on a Micromeritics ASAP 2010 or a TriStar II Plus porosimeter at 77 K (Micromeritics Instrument Corporation, Norcross, GA, USA). Samples were degassed to 1 µm Hg at 100 °C prior to sorption analysis. Surface area was calculated by the Brunauer-Emmett-Teller (BET) method, pore size by the Barrett-Joyner-Halenda (BJH) method, and total pore volume by the single point method at relative pressure (*P/P*_0_) 0.97.

Batch type experiments were used to characterize the binding capacity and affinity of the sorbent materials. Experiments were conducted in 20 mL scintillation vials (EPA Level 3; clear borosilicate glass; PTFE/silicone-lined cap) using a fixed mass of sorbent (indicated in text and figure captions). Target samples (10 mL) were prepared in 18.2 MΩ Milli-Q deionized water or in biodiesel synthesized in-house. Target solutions were added to the sorbents in the vials with a portion of the sample retained for use as a control during chromatographic analysis. Serial dilution of the retained sample was used for generation of a standard curve. Vials were incubated overnight on rotisserie mixers. Samples were filtered using 25 mm Acrodisc 0.2 μm syringe filters with PTFE membranes prior to processing and analysis. Difference method analysis was applied to determine the target removed from solution.

Analysis of glycerol was based on the method described by ASTM D6584-13 [27]. For initial characterization, all samples and control solutions were aqueous; water was removed (5 mL) using a Centrifan PE-T (Model 78-0070, KD Scientific, Holliston, MA, USA). A stock solution of N-methyl-N-(trimethylsilyl)trifluoroacetamide (MSTFA; 160 μL, Gelest, Inc., Morrisville, PA, USA) was added to the dried samples followed by incubation for 20 min. Heptane (1.6 mL) was then added to the samples, and they were transferred to autosampler vials for GC analysis. GC-MS analysis was performed using a Shimadzu GCMS-QP2010 (Shimadzu Scientific, Columbia, MD, USA) with AOC-20 autoinjector equipped with a Restex Rtx-5 (30 m × 0.25 mm ID × 0.25 μm df) cross bond 5% diphenyl 95% dimethyl polysiloxane column. A GC injection temperature of 200 °C was used with a 1:1 split ratio at a flow rate of 3.6 mL/min at 69.4 kPa. The oven gradient ramped from 50 °C (1 min hold time) to 180 °C at 15 °C/min and then to 300 °C at 20 °C/min where it was held for 5 min. For samples prepared in biodiesel, no water removal was necessary. A sample of the solution (100 μL) was added to MSTFA (100 μL). All other aspects of the protocol were identical to that described for samples in aqueous solution.

### 3.7. Biodiesel Synthesis

Biodiesel for sorbent analysis was prepared using food grade canola oil purchased at a local supermarket. Food grade canola oil (1 L) was warmed to 50 °C on a hotplate. In a separate container, methanol (355 mL) and sodium hydroxide (3.5 g) were mixed. This mixture was slowly added to the warmed oil, and the result was stirred for 2 h (50 °C). The reacted solution was moved to a separatory funnel and allowed to settle so that bottom layer of glycerol could be drained. Rotary evaporation was used to remove residual methanol from the final solution and the pH was verified to be neutral. This biodiesel preparation was used for evaluation of the sorbent materials. The biodiesel was spiked with glycerol to produce a 54 mM solution. Dilutions of this solutions using the biodiesel preparation provided additional concentrations for use in sorbent evaluations. These dilutions were also used to generate a standard curve. As described for the aqueous samples, target solutions were added to the sorbents in vials. Vials were incubated overnight on rotisserie mixers. Samples were filtered using 25 mm Acrodisc 0.2 μm syringe filters with PTFE membranes prior to processing and analysis. Difference method analysis was applied to determine the target removed from solution using a modification of the protocol described in Section 3.6. A 100 μL aliquot was added to 100 μL MSTFA. This solution was mixed well and incubated at room temperature for 20 min before addition of heptane (8 mL). The result was transferred to an auto sampler vial for analysis by the GC-MS protocol described in Section 3.6.

## 4. Conclusions

This study examined the potential for utilization of hierarchical organosilicate materials for the capture of glycerol as a representative biodiesel contaminant. Sulfonation of the sorbents promotes hydrogen bonding interactions and provides surface-target interactions similar to those of several commercially available sorbent materials, for example, Amberlite™ BD10DRY. The varied chemical compositions used in organosilicate sorbent synthesis provide differing points within the frameworks for sulfonation as well as varied total site content. This study demonstrated the importance of access to the total surface area for optimal performance characteristics. Higher affinity could be achieved through a greater degree of sulfonation, but performance suffered due to a loss in access to the total surface of the sorbent associated with morphological collapse. D13-Ph2(5) provides a compromise between degree of sulfonation and the resulting damage to sorbent morphology. This material provides the best per mass binding of the sorbents evaluated here in both aqueous solution and in biodiesel. The open morphology of the sorbents, in addition to providing binding site access, provides reduced resistance to flow when the materials are used in a column format [20,33]. This may prove useful for application relevant formats. Wood pulp–based materials like BD ZorbX offer extremely low backpressures but suffer from the resulting short contact times and low saturation capacities.

Ongoing work with these sorbents is focused on their potential for binding other classes of contaminants from biodiesel such as fatty acids and surfactants. Binding of multiple targets is possible using the sorbents described here; some preliminary data on the binding of surfactants from biodiesel has been collected. This additional capability is similar to that of the commercial sorbents described in the introduction. Binding of targets other than glycerol could reduce binding of this target. However, surfactants would be expected to occur at significantly lower concentrations than that of glycerol and would likely have minimal impact. Designing an effective dry wash media based on these organosilicate sorbents would likely require a combination of sorbents providing glycerol capacity as well as capacity for catalysts and surfactants. Increase of production scale will also be necessary for any applications. The materials described here are synthesized on the single gram scale. We have scaled synthesis of the ethane-diethylbenzene sorbents (ED11) to produce tens of ggrams simply by increasing the capacity of vessel utilized. Ongoing work seeks to develop protocols for larger scale production and to identify considerations of import to commercial scale synthesis.

The introduction mentions the potential for cost offset through recovery and repurposing of the glycerol byproduct. This effort did not focus on purification of glycerol. However, we have previously demonstrated the use of this type of sorbent in solid phase extraction for increasing the concentration of perchlorate and nitroenergetic targets [20,25,29,33]. In these cases, the target is adsorbed from aqueous solution and eluted from the sorbent using a solvent or an acid containing solution. Elution of glycerol from a sorbent column would likely require the use of a salt containing aqueous solution or a mixture of solvents. This process would not necessarily produce purified glycerol: the eluent would be expected to contain the mixture of targets that were adsorbed by the materials. Research into this potential application as well as scale up and capture of additional targets is ongoing.

## Figures and Tables

**Table 1:** Sorbents described under this study.

Scaffold Description	Material	Type	Grafted Phenyl Groups	Sulfonation
Silicate sorbent based on tetramethyl orthosilicate, 0.65 g mesitylene used in synthesis	S65	--	No	none
	S65-Ph(1)	1	Yes	standard
Silicate sorbent based on tetramethyl orthosilicate, 0.85 g mesitylene used in synthesis	S85	--	No	none
	S85-Ph(1)	1	Yes	standard
Ethane-bridged organosilicate, 0.5 g mesitylene used in synthesis	E25	--	No	none
	E25-Ph(2)	2	Yes	standard
Ethane-bridged organosilicate, 1.0 g mesitylene used in synthesis	E50	--	No	none
	E50-Ph(2)	2	Yes	standard
Organosilicate with mixed ethane and diethylbenzene bridging groups	ED11	--	No	none
	ED11-Ph	--	Yes	none
	ED11a(4)	4	No	standard
	ED11-Ph1(3)	3	Yes	standard
	ED11-Ph2(3)	3	Yes	standard
Diethylbenzene-bridged organosilicate	D13	--	No	none
	D13a(6)	6	No	standard
	D13b(6)	6	No	dilute
	D13c(6)	6	No	standard
	D13-Ph1(5)	5	Yes	standard
	D13-Ph2(5)	5	Yes	dilute
Divinylbenzene resin, synthesized with water	PDVB-2	--	No	standard
Divinylbenzene resin, synthesized with no water	PDVB-3	--	No	standard

**Table 2:** Morphological characteristics for the sulfonated sorbents.

Material	Type	Surface Area (m^2^/g)	Pore Volume (cm^3^/g)	Pore Diameter (Å)	*q_s_*		*k* (1/mg)	*n*	Chi^2^	Std. Error
					mg/g	μg/m^2^				
S65 †	--	591	0.675	65	--	--	--	--	--	--
S85 †	--	552	0.716	75	--	--	--	--	--	--
S65-Ph(1)	1	525	0.542	56	37.3	72.4	2.78	1	0.334	0.160
S85-Ph(1)	1	578	0.626	64	24.5	42.4	1.92	1	0.008	0.024
E25 †	--	1088	1.32	88	--	--	--	--	--	--
E50 †	--	1143	1.050	76 *	--	--	--	--	--	--
E25-Ph(2)	2	803	0.740	64	12.0	14.9	4.78	1	0.018	0.361
E50-Ph(2)	2	883	0.664	-- *	5.2	5.90	10.8	1	0.023	0.042
ED11 †	--	711	0.817	55	38.8	55.4	0.461	1	0.001	0.012
ED11-Ph †	--	498	0.560	57	--	--	--	--	--	--
ED11a(4)	4	87	0.111	55	18.0	207	0.920	1	0.022	0.047
ED11-Ph1(3)	3	147	0.151	48	21.3	145	13.5	1	0.057	0.069
ED11-Ph2(3)	3	27	0.047	50	12.2	452	24.0	1	0.007	0.031
D13 †	--	427	0.642	74	0.006	13.7	0.011	1	0.019	0.014
D13a(6)	6	3.1	0.005	-- *	16.4	5290	4.56	1	0.031	0.016
D13b(6)	6	337	0.536	75	273	810	1.96	1	0.001	0.008
D13c(6)	6	3.2	0.011	-- *	16.6	5188	4.45	1	0.016	0.037
D13-Ph1(5)	5	4.0	0.007	-- *	10.8	2700	3.95	1	0.081	0.082
D13-Ph2(5)	5	310	0.497	75	552	1780	4.15	1	0.058	0.020
PDVB-2	--	405	0.564	126	4.7	11.6	26.8	1	0.022	0.028
PDVB-3	--	336	0.375	65	11.2	33.3	15.8	1	0.277	0.141
BD-ZorbX γ	--	0.48	<0.01	--	4.8	--	6.01	1	0.021	0.046
DW-R10 γ	--	--	--	--	2.8	--	7.20	1	0.007	0.031

* Broad, no well-defined peak in the pore size distribution; † Sorbents with no sulfonation; γ Commercial resins.

## Supplementary Materials

The following are available online at www.mdpi.com/1996-1944/10/6/682/s1: Figure S1: Glycerol binding isotherms for Type 1 and Type 2 materials; Figure S2: Glycerol binding isotherms for Type 3 and Type 4 materials; Figure S3: Glycerol binding isotherms for Type 5 and Type 6 materials; Figure S4: Glycerol binding isotherms for non-sulfonated sorbents; Figure S5: Glycerol binding isotherms for poly(divinylbenzene) sorbents; Figure S6: Glycerol binding from biodiesel by developed materials; Figure S7: Glycerol binding from biodiesel by poly(divinylbenzene) materials; Figure S8: Glycerol binding from biodiesel by commercial resins.
