# Supplementary material for: Improving Sorbents for Glycerol Capture in Biodiesel Refinement
Source: Materials (Basel). 2017 Jun 21;10(6):682. doi: 10.3390/ma10060682 (PMC5554063; doi:10.3390/ma10060682)

materials

Supporting Information

Improving Sorbents for Glycerol Capture in Biodiesel Refinement

Brandy J. Johnson [1,*], Brian J. Melde [1,†], Martin H. Moore [1], Anthony P. Malanoski [1], Jenna R. Taft [2]

1 Center for Bio/Molecular Science and Engineering, Naval Research Laboratory, Washington, DC, USA; E-Mails: brandy.white@nrl.navy.mil (BJJ); martin.moore@nrl.navy.mil (MHM); brian.melde@nrl.navy.mil (BJM); anthony.malanoski@nrl.navy.mil (APM)

2 formerly, NOVA Research Incorporated, Alexandria, VA 22308 USA: jennartaft@gmail.com
* Correspondence: brandy.white@nrl.navy.mil; Tel.: +1-202-404-6100

Academic Editor: name
Received: date; Accepted: date; Published: date

This supporting information provides additional glyercol binding isotherms.

Figure S-1. Glycerol binding isotherms and data sets for the Type 1 and 2 sorbents developed under this study. Sorbent identified by label within the frame. Mass of sorbent (mg) used indicated by number on the isotherm (2.5 to 40 mg).

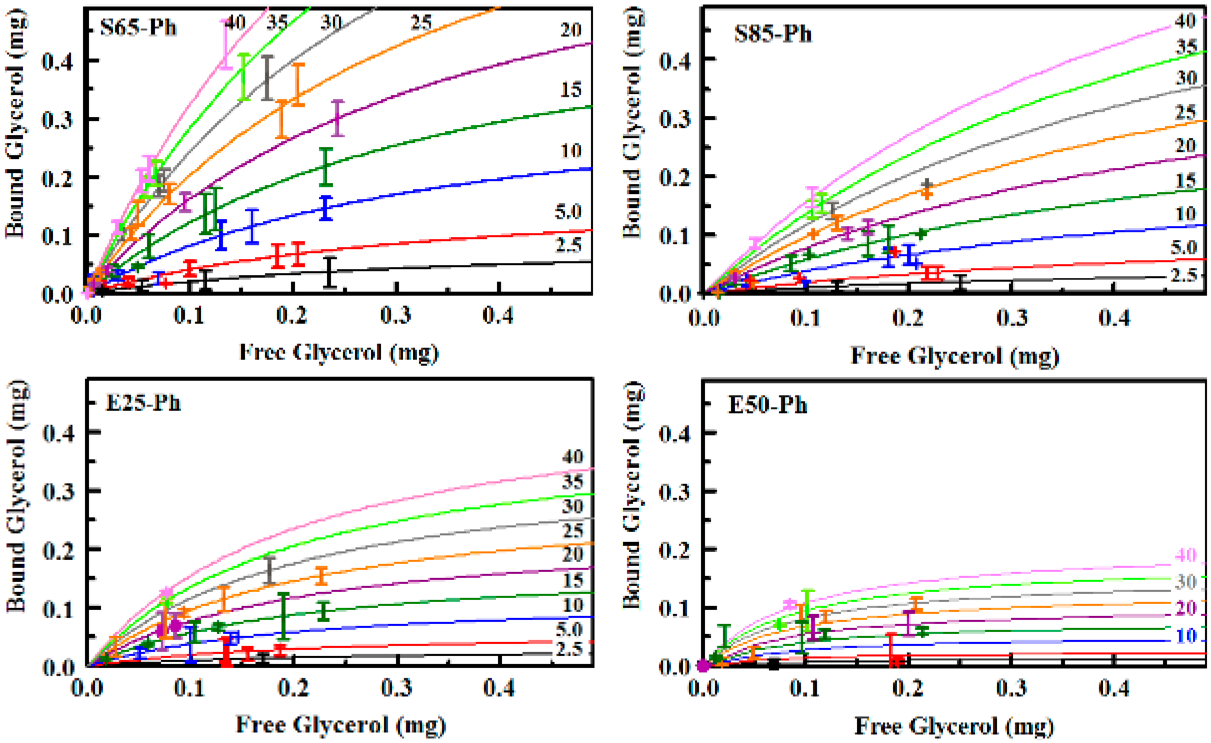

Figure S-2. Glycerol binding isotherms and data sets for the Type 3 and 4 sorbents developed under this study. Sorbent identified by label within the frame. Mass of sorbent (mg) used indicated by number on the isotherm (2.5 to 40 mg).

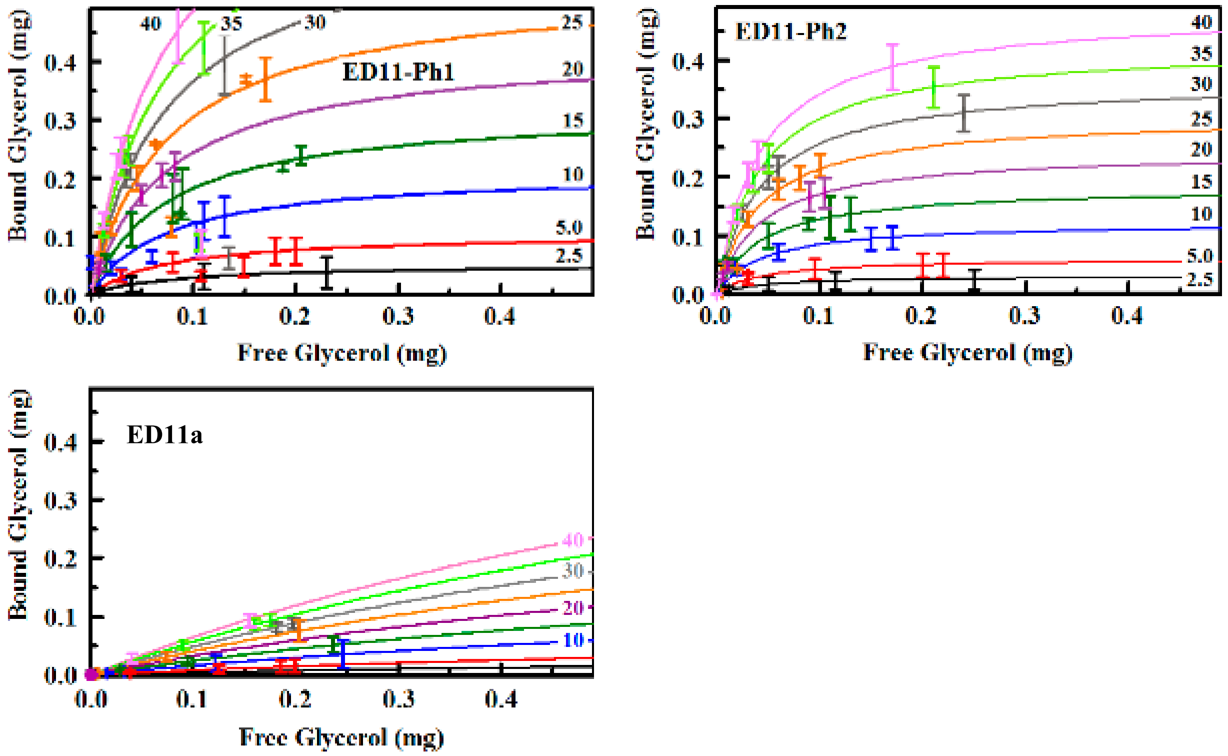

Figure S-3. Glycerol binding isotherms and data sets for the Type 5 and 6 sorbents developed under this study. Sorbent identified by label within the frame. Mass of sorbent (mg) used indicated by number on the isotherm (2.5 to 40 mg).

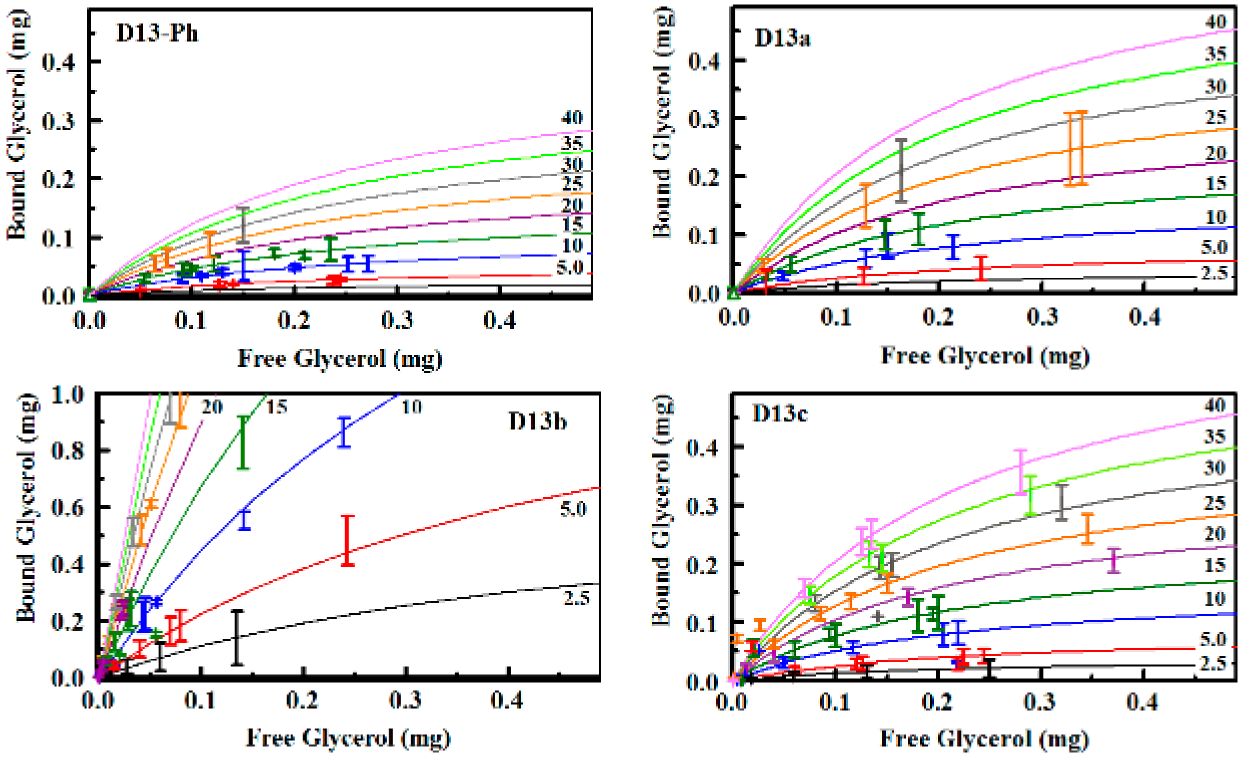

Figure S-4. Glycerol binding isotherms and data sets for non-sulfonated sorbents used for comparisons under this study. Sorbent identified by label within the frame. Mass of sorbent (mg) used indicated by number on the isotherm (2.5 to 40 mg).

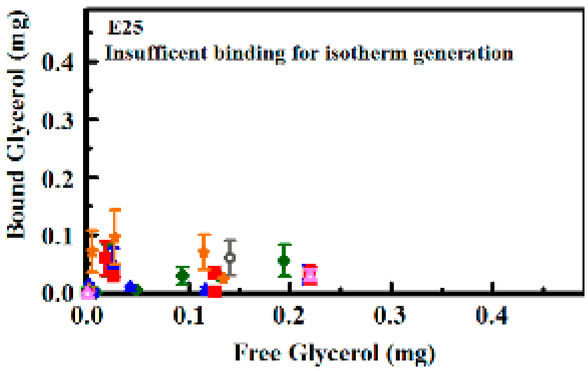

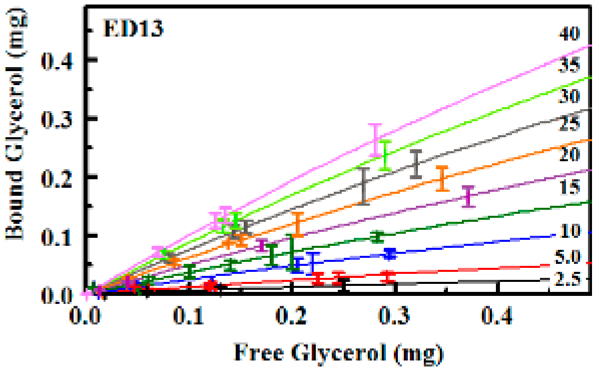

Figure S-5. Glycerol binding isotherms and data sets for poly(divinylbenzene) sorbents used for comparisons under this study. Sorbent identified by label within the frame. Mass of sorbent (mg) used indicated by number on the isotherm (5 to 30 mg).

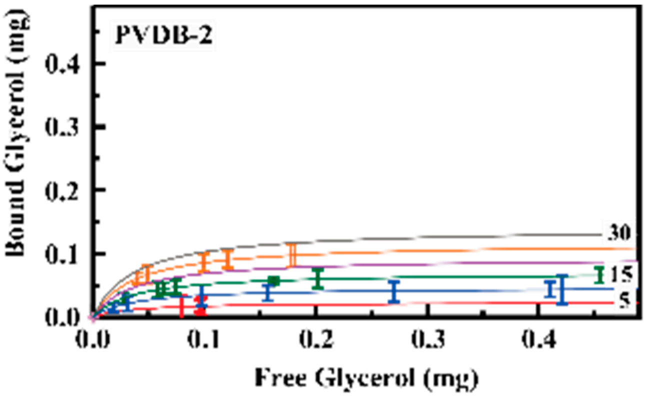

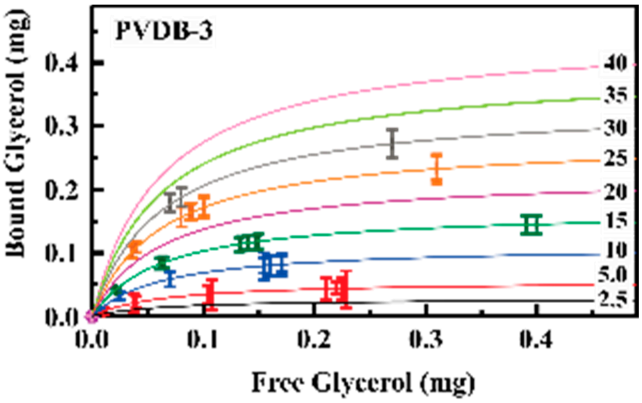

Figure S-6. Binding of glycerol from biodiesel by the sorbents developed under this study. Sorbent identified by label within the frame. Mass of sorbent (mg) used indicated by number on the isotherm (5 to 50 mg). Isotherms are those fitted for glycerol binding from aqueous solution (Table 1).

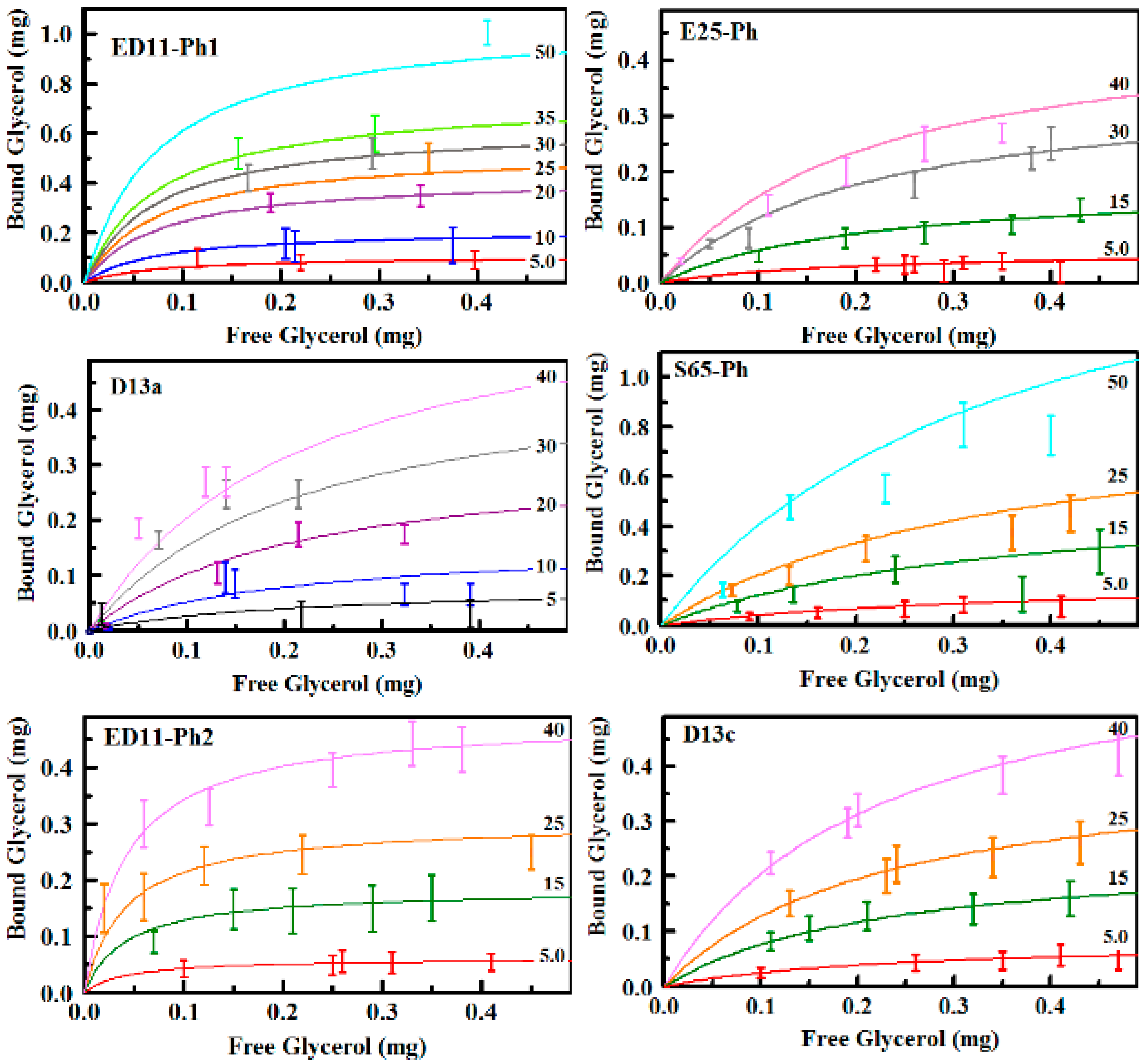

Figure S-7. Binding of glycerol from biodiesel by the poly(divinylbenzene) materials. Sorbent identified by label within the frame. Mass of sorbent (mg) used indicated by number on the isotherm (5 to 50 mg). Isotherms are those fitted for glycerol binding from aqueous solution (Table 1).

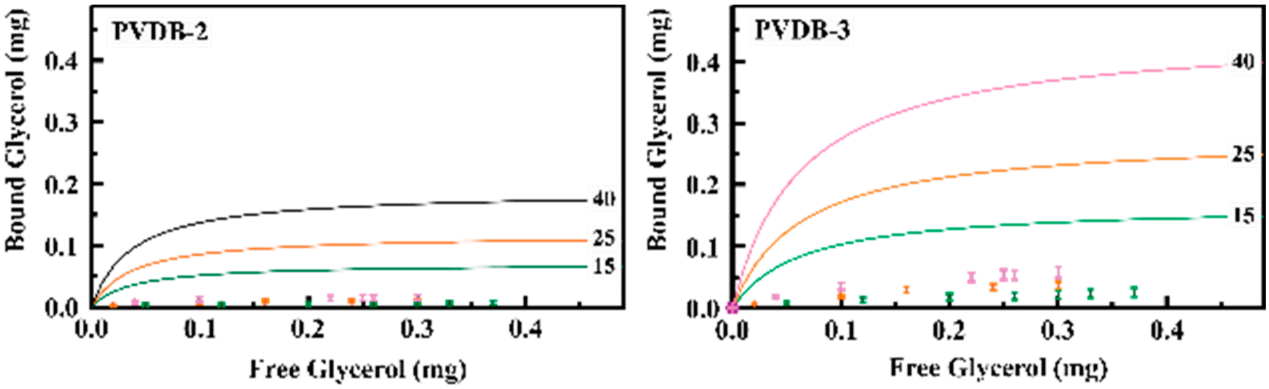

Figure S-8. Glycerol binding isotherms and data sets for the commercial resins used for comparisons under this study. Sorbent identified by label within the frame. Mass of sorbent (mg) used indicated by number on the isotherm (5 and 30 mg).

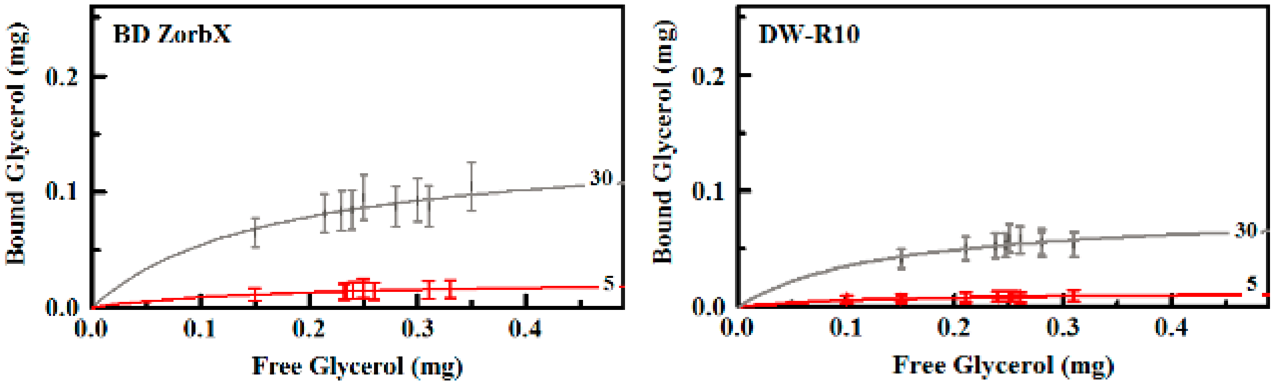

Figure S-9. Glycerol binding from biodiesel for the commercial resins used for comparisons under this study. Sorbent identified by label within the frame. Mass of sorbent (mg) used indicated by number on the isotherm (5 and 30 mg).

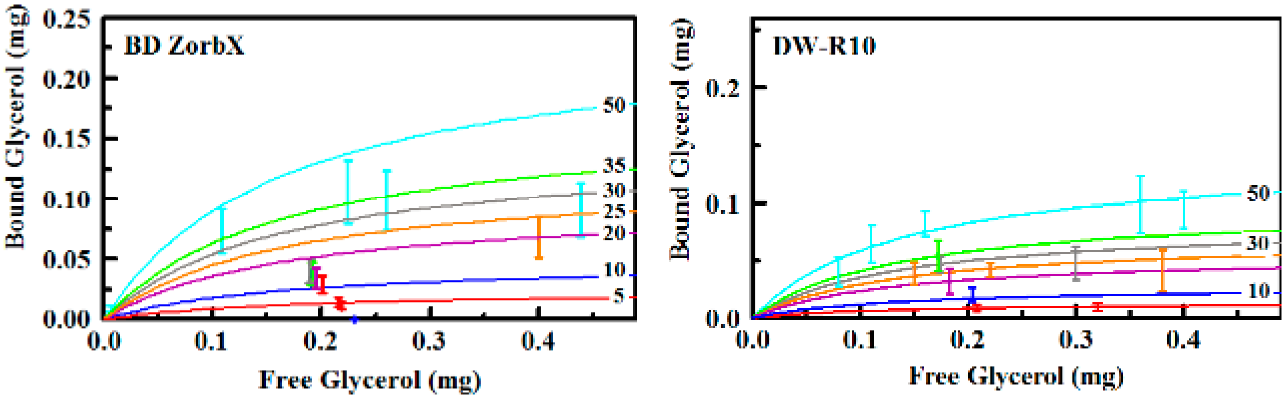

Supplement: Supplementary file 1 [file materials-10-00682-s001.pdf]
